# Alopecia areata during dupilumab therapy: 2 cases and review of the literature

**DOI:** 10.1016/j.jdcr.2026.04.024

**Published:** 2026-04-21

**Authors:** Amnah Nasser Almulhim, Hadeel Khalid Alotaibi, Saad Mohammad Alfehaid, Jury Waleed Albaker, Shahad Almakhaitah, Lenah Yassir Shaikh

**Affiliations:** a Consultant Dermatologist, Department of Dermatology, King Fahad Specialist Hospital–Dammam (KFSH-D), Dammam, Saudi Arabia; b Attachment Resident, College of Medicine, Imam Abdulrahman Bin Faisal University, Dammam, Saudi Arabia; c Service Resident, Department of Dermatology, Dammam Medical Complex, Dammam, Saudi Arabia; d Medical Student, College of Medicine, Imam Abdulrahman Bin Faisal University, Dammam, Saudi Arabia; e Medical Student, College of Medicine, King Faisal University, Al-Ahsa, Saudi Arabia

**Keywords:** alopecia areata, atopic dermatitis, chronic rhinosinusitis with nasal polyposis, dupilumab, paradoxical reaction.

## Introduction

Alopecia areata (AA) is a common immune-mediated nonscarring characterized by CD8+ T-cell-mediated follicular inflammation and predominantly T helper type 1 (Th1) cytokine profile.^1^ AA frequently coexists with atopic dermatitis (AD), complicating interpretation of disease onset or relapse during biologic therapy.

Dupilumab is a fully human monoclonal antibody targeting interleukin-4 receptor alpha subunit, thereby inhibiting interleukin (IL)-4 and IL-13 signaling within the Th2 pathway.^2^ Although dupilumab is effective for AD, asthma, and chronic rhinosinusitis with nasal polyposis, its effects on AA remain heterogenous.^3^ Both improvement and paradoxical induction or exacerbation of AA have been reported.^4^, ^5^, ^6^, ^7^, ^8^, ^9^, ^10^, ^11^, ^12^, ^13^, ^14^, ^15^, ^16^

We described 2 patients with prior AA who developed disease exacerbation or reactivation following dupilumab initiation and review the relevant literature.

## Case report

### Case 1

A 48-year-old man with bronchial asthma and chronic rhinosinusitis with nasal polyposis presented with progressive patchy hair loss involving the beard and mustache. His history included recurrent self-limited episodes of beard AA over 5 y. He had undergone 2 functional endoscopic sinus surgeries and recently required systemic corticosteroid therapy for chronic rhinosinusitis with nasal polyposis exacerbations.

Dupilumab was initiated due to corticosteroid-refractory disease. Four weeks after treatment initiation, the patient developed worsening and multifocal beard alopecia, differing in severity and persistence from prior episodes. There was no personal or family history of autoimmune diseases other than AA.

**Fig 1:**
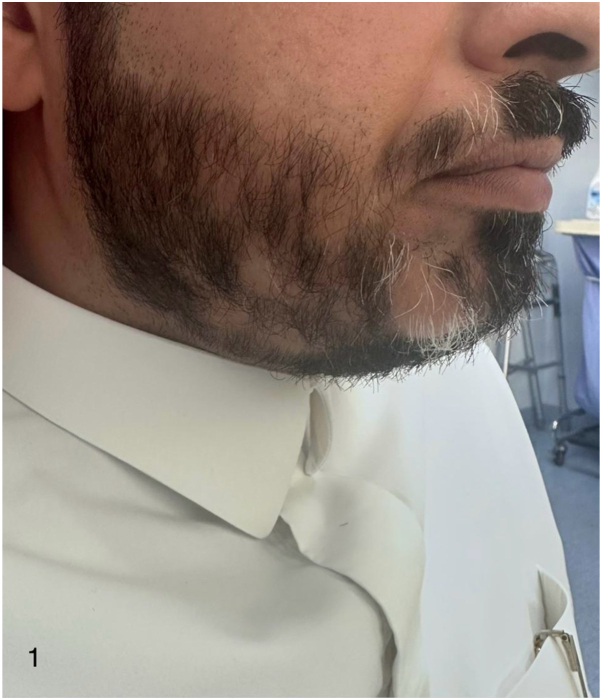
Case 1 – Alopecia areata over beard. Clinical photograph demonstrating multifocal patchy alopecia of the beard with sharply demarcated areas of hair loss prior to dupilumab discontinuation.

Physical examination demonstrated patchy, nonscarring alopecia of the beard and mustache (Fig 1). Dermoscopy revealed yellow dots, vellus hairs, and exclamation-mark hairs, consistent with active AA. Beard involvement was estimated 23.3%, using modified severity of alopecia tool score. Nail examination was normal. Laboratory evaluation, including thyroid function tests, autoimmune markers, and nutritional indices, was unremarkable.

**Fig 2:**
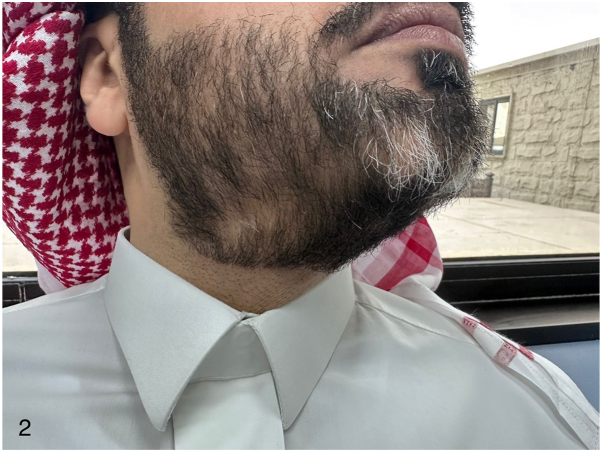
Case 1 – Beard regrowth after dupilumab discontinuation. Clinical photograph showing continued regrowth of terminal and vellus hairs following cessation of dupilumab therapy.

The patient was treated with intralesional triamcinolone (2.5 mg/mL), later increased to 5 mg/mL along with topical minoxidil. Despite 4 treatment sessions, disease progression continued with minimal growth. Following multidisciplinary discussion, dupilumab was discontinued and therapy was transitioned to mepolizumab. Marked hair regrowth was observed within 2 wk of dupilumab cessation, supporting a temporal association between dupilumab exposure and AA exacerbation (Fig 2).

### Case 2

A 41-year-old woman with severe treatment-refractory AD achieved significant improvement after dupilumab. Her medical history included thyroid carcinoma treated with complete thyroidectomy, and radioiodine ablation, and she was maintained on levothyroxine 25 μg daily. She reported a remote history of AA 10 y earlier that resolved with intralesional corticosteroid.

**Fig 3:**
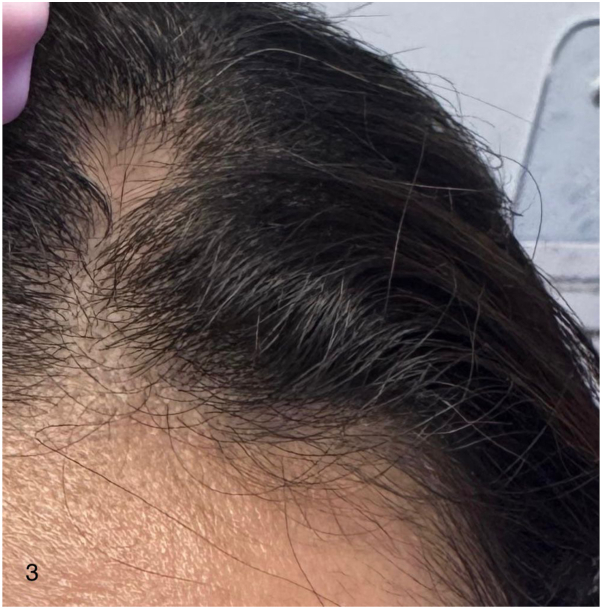
Case 2 – Scalp alopecia areata during dupilumab therapy. Clinical photograph demonstrating a localized patch of nonscarring alopecia areata involving the frontal scalp while continuing dupilumab therapy.

**Fig 4:**
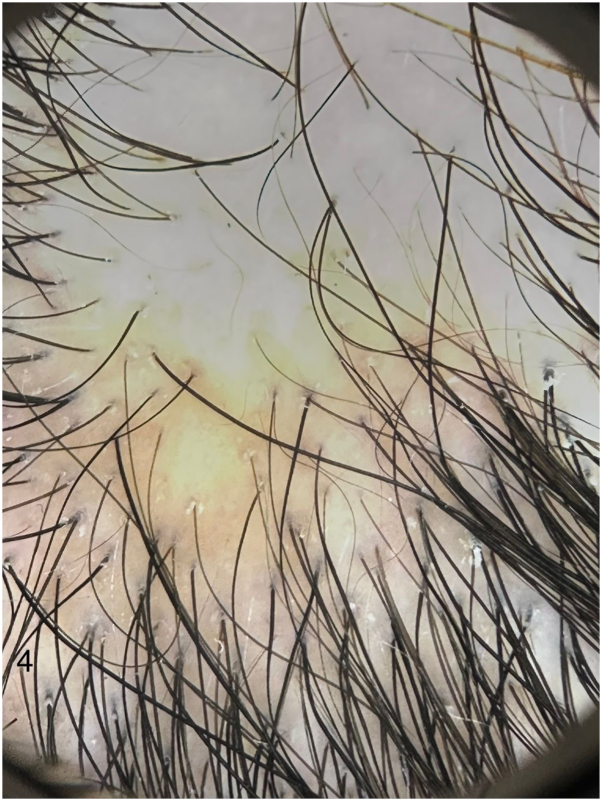
Case 2 – Dermoscopic findings of scalp alopecia areata. Dermoscopic image demonstrating features consistent with alopecia areata, including yellow dots and vellus hairs, while continuing dupilumab therapy.

**Fig 5:**
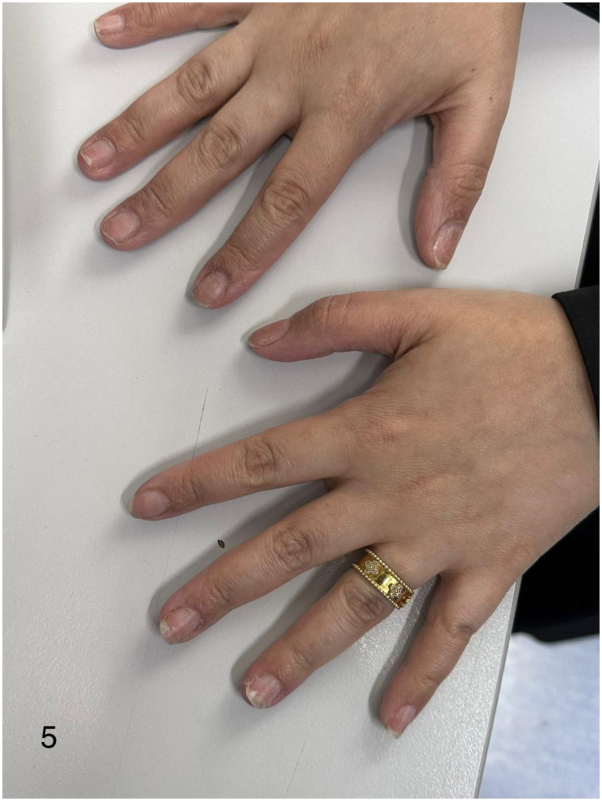
Case 2 – Nail changes. Clinical photograph demonstrating nail dystrophy with pitting, consistent with alopecia areata–associated nail involvement.

Approximately 18 wk after starting dupilumab, the patient noted a localized single patch of hair loss. Examination revealed a well-defined, nonscarring alopecic patch with minimal frontal hairline regression (Fig 3) corresponding to a severity of alopecia tool score of approximately 2%. Dermoscopy demonstrated vellus hairs consistent with AA (Fig 4). Nail examination revealed dystrophic changes with pitting (Fig 5).

Laboratory evaluation showed suppressed thyroid-stimulating hormone levels with normal free T3 and T4. Antinuclear antibodies and thyroglobulin IgG antibodies were elevated, while other laboratory results were unremarkable.

The patient was treated with intralesional triamcinolone (2.5 mg/mL). Given excellent AD control and limited AA involvement, dupilumab was continued. Complete regrowth occurred at initial site; however, a new alopecic patch subsequently developed, consistent with AA reactivation.

## Discussion

Immunologic mechanisms:

• The relationship between dupilumab therapy and AA remains incompletely defined.
• AD is predominantly Th2-driven, whereas AA is primarily mediated by Th1/interferon-γ signaling, with additional involvement of Th2 and Th17 pathways.^1^^,^^3^
• Selective Th2 blockade may theoretically shift immune balance toward Th1-dominant pathways, potentially triggering AA in predisposed individuals.

Potential contributing factors in the presented cases:

• AA is characterized by a relapsing–remitting course, and disease flares may occur independently of therapeutic interventions, particularly in patients with a prior history of AA.
• Case 1: Recent systemic corticosteroid exposure and subsequent tapering for chronic rhinosinusitis with nasal polyposis may have altered immune equilibrium and contributed to disease activation. Chronic airway inflammation and cumulative disease burden may also act as nonspecific inflammatory or stress-related triggers.
• Case 2: The presence of autoimmune thyroid disease and serologic autoimmunity suggests an underlying predisposition to immune dysregulation.
• Although the contribution of concomitant medications or comorbidities cannot be entirely excluded, the temporal relationship between dupilumab initiation and AA onset, along with rapid clinical improvement following drug discontinuation in Case 1, supports a potential treatment-related association.

Evidence from the literature:

• A recent systematic review reported both improvement and paradoxical AA reactions during dupilumab therapy, with differing times to onset.^17^
• Population-based analyses have demonstrated an increased risk of AA in dupilumab-treated patients beyond 16 wk of therapy.^18^
• Early immune modulation may explain AA exacerbation occurring within weeks of treatment initiation, as observed in our first case.

Clinical outcomes:

• Clinical outcomes are variable.
• Some patients experience regrowth despite continuation of dupilumab, whereas others require drug discontinuation to achieve improvement.
• This heterogeneity suggests immune dysregulation rather than direct drug toxicity as the underlying mechanism.

Reported cases:

• A review of the literature identified 17 reported cases of AA associated with dupilumab use,^7^, ^8^, ^9^, ^10^, ^11^, ^12^, ^13^, ^14^, ^15^, ^16^^,^^19^ summarized in Table I.

**Table I:** Summary of reported cases of alopecia areata associated with dupilumab therapy

Reference	Sex	Age	History of AA	Onset after dupilumab	Morphology/site of AA after dupilumab	Histopathology	Dupilumab stopped?	Treatment/Outcome
Kanda 2019	Male	35	No	6 wks	Patchy, frontal/occipital/bitemporal scalp	NA	No	methylprednisolone (0.5 g/d) for 3 d → 78% improvement
Barroso-García 2018	Male	31	No	6 wks	Patchy, anterior scalp	Perifollicular infiltrate, fibrotic tracts, exocytosis, spongiosis, parakeratosis	No	IL TAC; outcome not reported
Carnicle 2019	Female	42	Yes	16 wks	Diffuse, scalp + pubic area	NA	No	IL TAC → near-complete regrowth
Barbarin 2019	Female	23	No	48 h	Multiple alopecia patches, frontal/vertex/occipital scalp	NA	Yes	Stopped dupilumab → full regrowth
Flanagan 2019	Male	27	No	18 wks	Diffuse nonscarring alopecia crown/temporal scalp	Hair miniaturization, peribulbar inflammation, severe sebaceous gland atrophy	Yes	Stopped dupilumab + IL TAC → full regrowth
Koo 2025 - Case 1	Male	28	No	8-12 wks	Erythematous scaly alopecia patches, parietal scalp	NA	No	Continued dupilumab → spontaneous regrowth
Koo 2025 - Case 2	Male	47	No	8-12 wks	Erythematous scaly alopecic patches	NA	No	Continued dupilumab → spontaneous regrowth
Koo 2025 - Case 3	Male	25	No	8 wks	Alopecia with frontal hairline erythema	Parakeratosis, acanthosis, spongiosis, perivascular infiltrate	Yes	Stopped dupilumab + steroids/cyclosporine → regrowth
Chung 2019 - Case 1	Female	51	No	26-30 mo	Generalized thinning → discrete alopecia patches	NA	No	Resumed dupilumab monthly → 90% regrowth
Chung 2019 - Case 2	Male	25	Yes	6-8 wks	alopecia totalis with loss of the few remaining hair patches over the scalp	NA	Yes	Stopped dupilumab → tofacitinib → improvement within 6 wks of treatment
Mitchell 2018	Male	29	No	5 wks	Patchy, posterior scalp	NA	No	IL TAC → gradual improvement
Salguero-Fernandez 2018	Male	33	No	7 wks	Diffuse alopecia, frontal/occipital scalp + beard	Spongiosis, parakeratosis, peribulbar infiltrate	No	Continued dupilumab + mometasone → regrowth
Maiolini 2021	Male	22	No	22 wks	Erythematous scaling alopecia, plaque/vertex scalp	Psoriasiform dermatitis, parakeratosis, spongiosis	No	Betamethasone + calcipotriol → complete regrowth
Maloney 2019 – Case 1	Male	29	No	5 wks	Patchy scalp AA, no erythema/scaling (SALT 20)	NA	No	IL TAC → slow improvement
Maloney 2019 – Case 2	Male	27	No	18 wks	Patchy scalp AA with erythema/scaling (SALT 20)	Sebaceous gland atrophy + AA features	Yes	Stopped dupilumab + IL/topical steroids → regrowth within 8 wks
Maloney 2019 – Case 3	Male	33	No	7 wks	Patchy scalp & beard AA with dermatitis (SALT 42)	Spongiosis, parakeratosis, plasma cells + AA features	No	Continued dupilumab + topical mometasone → regrowth by 3 mo
Maloney 2019 – Case 4	Male	31	No	6 wks	Patchy scalp AA with dermatitis (SALT 4)	AA features + epidermal inflammation	No	IL TAC → outcome not reported

*AA,* Alopecia areata.
• Onset ranged from 6 to 18 wks after treatment initiation.
• Most cases involved patchy or diffuse nonscarring alopecia of the scalp, with variable response to continuation or discontinuation of therapy.
• Reactivation of preexisting AA appears less frequently reported than de novo disease.

Paradoxical reactions to biologic therapy:

• Paradoxical inflammatory reactions have been reported across multiple biologic therapies.
• Examples include eczematous eruptions induced by brodalumab in psoriatic patients that resolved after switching to risankizumab, and paradoxical hidradenitis suppurativa triggered by adalimumab biosimilars and successfully treated with guselkumab.
• Collectively, these observations support the concept that targeted cytokine blockade may unmask alternative immune pathways rather than reflect direct drug toxicity.^20^

Clinical implications:

• Clinicians should remain vigilant for AA development or relapse in patients receiving dupilumab, particularly those with a personal history of AA.
• Continuation of therapy may remain appropriate when overall quality-of-life benefits outweigh the severity of AA, emphasizing the need for individualized decision-making.
• In patients with mild or limited disease, continuation of dupilumab combined with topical treatment for AA, as observed in Case 2, may be appropriate.
• In contrast, as demonstrated in Case 1, patients with severe or painful hair loss may benefit from discontinuation of dupilumab or switching to an alternative biologic after discussion within a multidisciplinary team.
• Paradoxical inflammatory reactions have been reported with various biologic therapies, highlighting the importance of appropriate patient counseling and close clinical monitoring during treatment.^20^

## Conclusion

These cases highlight early exacerbation and delayed reactivation of AA during dupilumab therapy in patients with a prior history of disease. Awareness of this potential association is important for counseling, monitoring, and individualized management of patients receiving dupilumab.

## Conflicts of interest

None disclosed.
